# First glycopeptide-resistant Enterococcus faecium isolate from blood culture in Ankara, Turkey.

**DOI:** 10.3201/eid0701.010128

**Published:** 2001

**Authors:** A Basustaoglu, H Aydogan, C Beyan, A Yalcin, S Unal


infections and disseminated disease involving multiple organs in immunocompromised patients. In such cases the disease can mimic septicemic melioidosis (4,5).

In this previously healthy patient, infection probably originated from the facial abscess. The patient was negative for HIV antibody (Serodia), had no history of diabetes mellitus or other compromising illnesses, and had no evidence of immunodeficiency. In a previous case of disseminated *C. violaceum* infection in a young patient, postmortem findings revealed numerous cortical infarcts and hemorrhages (6). Our isolate from a brain abscess is yet another case of a relatively avirulent saprophytic microorganism resulting in a deep-seated infection in a well-nourished, previously healthy person.

**Dhammika Nanda Atapattu,***
**Dhammika Priyal Jayawickrama,*† and**
**Vasanthi Thevanesam***
*University of Peradeniya, Peradeniya, Sri Lanka;
†General Hospital, Kandy, Sri Lanka

### References

1. Mathisen GE, Johnson JP. Brain abscess. Clin Infect Dis 1997;25:763-81.
2. Mandell GL, Bennett J, Dolin R. Principles and practice of infectious diseases. 4th ed. New York: Churchill Livingstone; 1995: p. 887-99.
3. Stokes EJ, Ridgway GL, Wren MWD. Clinical microbiology. 7th ed. London: Edward Arnold; 1993: p. 239-50.
4. Murray PR, Baron EJ, Pfaller MA, Tenover FC. Manual of clinical microbiology. 6th ed. Washington: ASM press; 1995: p. 503.
5. Mitchell RG. In: Parker MT, Duerden BI, editors. Miscellaneous bacteria. Topley and Wilson's principles of bacteriology, virology and immunity, Vol. 2. 8th ed. London: Edward Arnold; 1990: p. 589-91.
6. Ti TY, Tan WC, Chong APY. Non fatal and fatal infections caused by *Chromobacterium violaceum*. Clin Infect Dis 1993;17:505-7.

### First Glycopeptide-Resistant *Enterococcus faecium* Isolate from Blood Culture in Ankara, Turkey

**To the Editor:** Glycopeptide-resistant enterococci infections are a major problem in hospitals. Infection or colonization by vancomycin-resistant enterococci was first reported in France (1) and the United Kingdom (2); since then, these organisms have been reported throughout the world. In Turkey, vancomycin and teicoplanin have been used to treat serious methicillin-resistant *Staphylococcus aureus* and ampicillin-resistant enterococci infections.

We describe the case of an acute myelocytic leukemia patient with vancomycin-resistant enterococci bloodstream infection. This is the first glycopeptide-resistant *Enterococcus faecium* isolate from our hospital and from Ankara, Turkey. The patient had not been cared for at another institution.

A 68-year-old man, hospitalized with acute myelocytic leukemia, had fever episodes during the neutropenia following three courses of remission-induction chemotherapy (daunorubicin+cytosine arabinoside). A combination of antibiotics including vancomycin, ceftazidime (sometimes imipenem), and amikacin was administered with different regimens during the 5 months of hospitalization. Blood, urine, and rectal swab cultures during this period were positive for different *Enterobacteriaceae* spp. but always negative for vancomycin-resistant enterococci. For long-term hospitalizations, our center routinely performs surveillance rectal swab cultures. At the end of month 5, *E. faecium* was isolated from the blood cultures, just 1 day before the patient's death.

The strain was identified by conventional methods, commercial automatic systems (API Strep-Biomerièux, France), and polymerase chain reaction. Susceptibility patterns showed that the isolate was resistant to all antibiotics except ciprofloxacin and levofloxacin. When the E-test was used, MIC levels for vancomycin, teicoplanin, ciprofloxacin, and levofloxacin were 256 µg/mL, 64 µg/mL, 0.75 µg/mL, and 1.5 µg/mL, respectively. *VAN-A1* and *Van-A2* type resistance genes were detected by polymerase chain reaction. Hacettepe University microbiology laboratories confirmed these results (3,4).

After this strain was isolated, 1,266 stool and 176 rectal swab samples were taken from hospital personnel in three sessions ≥1 week apart, and patients were tested for vancomycin-resistant enterococci. Swab cultures from all environmental surfaces (bed rails, bedside commodes, carts, charts, doorknobs, faucet handles) were also examined. We injected all samples with 5% sheep blood agar with vancomycin (6 mg/L); vancomycin-resistant *E. faecium* was not identified in any sample.

This was the first case of high-level vancomycin-resistant enterococci with a class A phenotype isolated from a person in our hospital or in Ankara, Turkey. To prevent the organism's spread, we implemented the recommendations of the Hospital Infection Control Practices Advisory Committee (5).

**Ahmet Basustaoglu,* Hakan Aydogan,* Cengiz Beyan,* Atilla Yalcin,* Serhat Unal†**
*Gülhane Military Medical Academy, Etlik Ankara, Turkey; †Hacettepe University, Ankara, Turkey

### References

1. Leclercq R, Derlot E, Duval J, Courvalin P. Plasmid-mediated resistance to vancomycin and teicoplanin in *Enterococcus faecium*. N Engl J Med 1988;319:157-61.
2. Uttley AH, George RC, Naidoo J, Woodford N, Johnson AP, Collins CH, et al. High level vancomycin-resistant enterococci causing hospital infection. Epidemiol Infect 1989;103:173-81.
3. Dutka-Malen S, Evers S, Courvalin P. Detection of glycopeptide resistance genotypes and identification of the species level of clinically relevant enterococci by PCR. J Clin Microbiol 1995;33:24-7.
4. Handwerger S, Skoble J, Discotto LF, Pucci MJ. Heterogeneity of the VanA gene clusters in clinical isolates of enterococci from the northeastern United States. Antimicrob Agents Chemother 1995;39:362-8.
5. Hospital Infection Control Practices Advisory Committee (HICPAC). Recommendations for preventing the spread of vancomycin resistance. Infect Control Hosp Epidemiol 1995;16:105.

### Antimicrobial-Drug Use and Methicillin-Resistant *Staphylococcus aureus*

**To the Editor:** We read with great interest the debate on the contribution of antimicrobial selection pressure to changes in resistance in *Salmonella enterica* serovar Typhimurium and the comparison made with methicillin-resistant *Staphylococcus aureus* (MRSA) (1).

We strongly agree with Davis et al. that infection control practices must play a central role in successful MRSA control programs. However, we disagree that the antimicrobial-drug use practices that contribute to the control of MRSA have not been scientifically defined. In a recent review, we identified more than 20 studies on consistent associations, dose-effect relationships, and concomitant variations, all supporting a causal relationship between antimicrobial-drug use and MRSA (2).

Since our review, seven other studies have reported on the contribution of antimicrobial-drug use to MRSA colonization and infection in patients, or to high MRSA rates in health-care settings (3-9). One study reports a decrease in the rate of new MRSA cases after major reduction in antimicrobial-drug use (5). Although a lower number of discharges and a shorter hospital stay recorded during the 2-year postintervention period have been proposed as other explanations (10), the sharp decrease in new MRSA cases after the new antibiotic formulary was implemented (a delay of only a few months) supports the hypothesis that reduced antimicrobial pressure contributed to the decline. Additionally, at the recent 4th Decennial International Conference on Nosocomial and Healthcare-Associated Infections, at least five reports addressed either (a) antimicrobial-drug use and increased MRSA incidence or (b) antimicrobial-drug use as an independent risk factor for MRSA acquisition or for persistent MRSA colonization after mupirocin treatment (11).

When antimicrobial classes are taken into account separately, cephalosporins and fluoroquinolones are often identified as risk factors for MRSA (2-5,8,11). The mechanisms that would explain the participation of these two classes are not fully understood. However, fluoroquinolones directly enhance the expression of high-level oxacillin-resistant *S. aureus* in vitro (11, p.202). Another recent study shows that sub-MIC levels of ciprofloxacin increase adhesion of quinolone-resistant MRSA (12), which could explain persistent MRSA colonization and failure of mupirocin treatment in patients who received a fluoroquinolone (11, p.197). MRSA outbreaks in surgical patients have been controlled by isolating patients and abandoning third-generation cephalosporins for surgical prophylaxis (3). As stated by Davis et al., dissemination of epidemic clones does not necessarily require antimicrobial selection pressure; however, the above studies suggest participation of antimicrobial drugs in MRSA colonization and outbreaks.

Finally, when citing Dutch infection control measures as an example of successful control of MRSA, Davis et al. omit the fact that, among European countries, the Netherlands has the lowest antimicrobial-drug use in primary health

